# Glycated Albumin Percentage is Correlated With HbA1c: Theoretic Marker in Patients With Altered Erythrocyte Turnover

**DOI:** 10.1177/19322968251384304

**Published:** 2025-10-21

**Authors:** Mats Gåfvels, Per Swärd, Ning Xu, Joel Svensson

**Affiliations:** 1 Department of Medical Sciences, Uppsala University Hospital, Uppsala, Sweden; 2 Clinical and Molecular Osteoporosis Research Unit, Departments of Orthopedics and Clinical Sciences, Skåne University Hospital, Lund University, Malmö, Sweden; 3 Division of Clinical Chemistry and Pharmacology, Department of Laboratory Medicine, Lund University, Lund, Sweden; 4 Unilabs Laboratory Medicine, Skaraborg Hospital, Skövde, Sweden

**Keywords:** hyperglycemia, glucose monitoring, HbA1c, hemoglobinopathy, glycated serum protein, glycated albumin

## Abstract

**Introduction:**

The gold standard for monitoring long-term glucose levels in patients with diabetes mellitus is glycated hemoglobin (HbA1c). In conditions where the erythrocyte half-life is decreased, for example, in long-term kidney diseases and hemoglobinopathies, HbA1C may underestimate the long-term glucose exposure. Therefore, in these patient groups, other methods to monitor long-term glucose have been suggested, including glycated serum protein (GSP). To further optimize the method, a correction against total serum albumin has been proposed, defined as a percentage of glycated albumin (%GA). The aim of this study was to investigate the correlation between HbA1c, GSP, and %GA—a strong correlation to HbA1c would strengthen the potential usefulness of GSP and %GA as alternative methods to monitor glucose exposure in certain patient populations.

**Methods:**

In this study, randomly collected human samples (n = 271), with different levels of HbA1c were analyzed for GSP and total serum albumin and a %GA was calculated. We also divided the samples into subgroups based on their HbA1c-result, age, and gender.

**Results:**

Both %GA and GSP were strongly correlated with HbA1c, where %GA displayed the strongest correlation (*R*^2^ 0.77 compared with *R*^2^ 0.66.). When dividing into subgroups based on HbA1c-results, statistically significant differences in %GA were observed between all the different subgroups.

**Conclusion:**

In conclusion, the findings of this study strengthen the possibility of using GSP and %GA as possible alternatives or at least a supplement to HbA1c for monitoring long-term glucose exposure. Theoretically, particularly %GA could have the potential to supplement HbA1C in patients where the erythrocyte half-life is altered.

## Introduction

Diabetes mellitus is a disease which imposes a major burden on the affected individual as well as on society and the prevalence is increasing worldwide. Living with diabetes mellitus is associated with a with a number of microvascular and macrovascular complications. Glycemic control is essential in the management of the disease and to reduce the risk of future complications. As an estimate of long-term glucose exposure, it is recommended to measure glycated hemoglobin (HbA1c), an estimate of the average blood glucose over the last two to three months.^1^ Glycated hemoglobin has also been described as a risk marker of future complications from hyperglycemic diseases.^2^

Glycated hemoglobin has furthermore been implemented as a diagnostic tool for diabetes mellitus. According to the World Health Organization (WHO), HbA1C ≥ 48 mmol/mol on two different occasions is required to fulfill the diagnosis criteria for diabetes mellitus in both Sweden and globally.^3^

However, the HbA1C analysis is considered to have limitations, especially among individuals with abnormal types of hemoglobin where the erythrocyte turnover is considerably affected.^4^ A considerably affected erythrocyte turnover can be expected in conditions such as hemoglobinopathies, thalassemia’s with hemolysis, long-term kidney disease, long-term malaria, and sickle cell disease. The International Federation of Clinical Chemistry and Laboratory Medicine (IFCC) standardization of the HbA1c analysis often uses chromatographic methods which in a considerable extension interfere and underestimate the HbA1c level in individuals with shortened life span of erythrocytes.^5^

Pathological variants of hemoglobin as observed in sickle cell disease including hemoglobin variants as hemoglobin SC carriers and also hemoglobin CC carriers have significantly lowered life span of erythrocytes, decreased to at least 75% of the normal erythrocyte life span. For these individuals, it is recommended to supplement HbA1c with alternative methods when estimating long-term glucose exposure.^6^ In other conditions with altered erythrocyte turnover, such as aplastic anemia, glycated serum protein (GSP), or glycated albumin are preferred as diagnostic tools for monitoring glucose homeostasis while HbA1c is not recommended.^7^

Also, in conditions such as gestational diabetes mellitus and diabetes mellitus type 1 with relatively rapid onset, there are limitations in the usefulness of HbA1c.^8,9^ It was recently shown that glycated albumin (GA) measured weekly after self-blood sampling in combination with self-review lifestyle behaviors might potentially benefit glycemic management in people with type 2 diabetes mellitus, with the advantage of faster feedback of glycemic monitoring from the GA test compared with the HbA1c test.^10^

Except hemoglobin, other serum proteins are subjected to irreversible glycation. This process is one kind of posttranslational modification where glucose is added nonenzymatically. The most highly abundant serum protein, albumin, is susceptible to glycation of multiple lysine residues.^11^ Glycated albumin is considered to account for about 90% of GSP. It has been observed that compared to hemoglobin, albumin reacts 10 times more rapidly with glucose. Thus, the onset of a hyperglycemic condition could be detected more rapidly based on the glycation status of albumin compared to HbA1c. Glycated serum protein reflects the glycemic status of an individual over a shorter time period—about two to three weeks.^12,13^

Proposed weaknesses of using GSP^10^ and the comparable fructosamine are that they may underestimate glucose exposure in conditions with altered levels of total albumin such as nephrosis, hypoalbuminemia, liver diseases as cirrhosis, and different kinds of inflammatory processes. These conditions result in lowered levels of total serum albumin because of lowered hepatic function, elevated renal albumin excretion, increased vascular leakage of albumin, and an inflammatory response which limits albumin synthesis. Theoretically, a deranged serum albumin homeostasis could confound the interpretation of GSP. By correcting for the total concentration of serum albumin, it would be possible to account for variations in serum albumin. The calculation of %GA has been described previously:^12^

$$\%\mathrm{GA}=\frac{\mathrm{GSP}\;(\mu \mathrm{mo}1\;L^{-1})\;\times \;0.182\;+\;1.97}{\mathrm{total}\;\mathrm{albumin(g}\;{\mathrm{dL}}^{-1})}\;+\;2.9$$

The aim of this study was to investigate the correlation between HbA1c, GSP, and %GA. Two cohorts with known HbA1c levels were used, one cohort with low HbA1c (<40 mmol/mol) and one cohort with high HbA1c (>56 mmol/mol). The selected cohorts reflect two distinct groups well below respectively well above the considered diagnosis limit, thereby ascertaining a considerable variability in long-term glucose exposure and providing the possibility to assess the %GA-assay performance in two divergent cohorts.

## Material and Methods

### Material

Samples were collected from the department of Clinical Chemistry, Skåne University Hospital, Malmö. Sweden. Ethical application was approved by the Swedish Ethical Review Authority (Ref no 2014/79). Samples were collected in Ethylenediaminetetraacetic acid tubes, 5 mL (EDTA) from Becton Dickinson Co., 4 mL of sample volume. Samples were collected consecutively from the routine HbA1c analysis during the date of 30/09/14 and between the dates of 03/06/15 until 10/06/15.

In total, 271 samples were collected during these dates. Since the samples were collected consecutively, they reflect the ordinary selection of samples being analyzed for HbA1c in a routine hospital laboratory, including a few individuals being under 18 years of age (n = 6). When division in different age subgroups was performed, we aimed to reach a reasonable even number of included individuals in each cohort. Initially, the samples were anonymized, and they were labeled with new serial numbers. After HbA1c levels in hemolysates (whole blood) had been analyzed within 1 hour, plasma was recovered after low-speed centrifugation (2000*g* for 10 min). All samples were included as distinct samples and no duplicates or individuals with double sampling were included. Analysis of HbA1c was performed within 1 h, and the plasma samples were analyzed for GSP and albumin within 48 h. When the samples were not handled, they were stored in a refrigerator (+4°C).

### HbA1c

Analysis of HbA1c was performed by Bio-Rad’s High Pressure Liquid Chromatography Variant™ ΙΙ Turbo and Hemoglobin A1c Kit–2.0 program (Department of Clinical Chemistry, Skåne University Hospital). The assay is continuously performed in the laboratory with the precision ratio of coefficient of variation (CV) <3% (level 36 mmol/mol) and the precision ratio of CV <3% (level 68 mmol/mol).

### GSP and Total Serum Albumin

The analysis of GSP was performed as described by the assay manufacturer Diazyme Laboratories.^12,13^ The method is an enzymatic assay in three steps, initiated with digestion of GSP into glycated protein fragments with low molecular weight. This is followed by Fructosaminase catalyzation and eventually a colorimetric end-point reaction where the absorbance in a specific span is proportional to the GSP concentration. Our analyses were performed in the routine laboratory of the department of clinical chemistry in Skåne University Hospital. This assay and the analysis of total serum albumin were both run on a Cobas c701 module (Roche Diagnostics, Basel, Switzerland). The GSP assay was validated according to local laboratory standards with within run precision evaluation of two control levels with 10 replicates each; control level 1, 204 µmol/L, with CV < 1.1% and control level 2, 751 µmol/L, with CV < 0.7%. The assay of total serum albumin is continuously performed in the laboratory with the precision ratio of CV < 1.8% (level 33 g/L and the precision ratio of CV <1.4% (level 56 g/L). The assays had been validated for human plasma and serum samples.^13,14^

### Statistics

Statistical analyses were performed with Microsoft Excel version 16.37 for section 3.1 including calculation of correlation coefficients. For sections 3.2 and 3.3 SPSS version 19.0 software (SPSS Inc., Chicago, IL) was used. *P* values were calculated with two-sample *t*-tests when comparing different means. The level of <.05 was considered as statistically significant.

## Results

### Overall GSP and %GA

In total, 271 consecutively collected samples during two periods were included. The samples were from individuals between 5 and 95 years of age. The mean age was 62.6 years. The number of females and males were 114 and 157, respectively. The mean HbA1c value was 52.6 mmol/mol. The mean %GA was 19.2%. The overall distribution of the results is presented in Figure 1. The %GA was strongly correlated to HbA1c, and the *R*^2^ was calculated to 0.77 (95% confidence interval [CI] 0.75-0.79). The GSP without correction for total albumin concentration showed a lower *R*^2^ calculated to 0.66 (95% CI 0.27-1). Using the equation to link %GA and HbA1c: y = 0.267x + 6.04 R² = 0.770 (Figure 1), the suggested upper cutoff at the HbA1c level 48 mmol/mol results in the cutoff level of %GA = 18.9 and for GSP the equation y = 4.8771x + 100.93 R² = 0.655 (Figure 1) with the cutoff at the HbA1c level 48 mmol/mol results in the GSP cutoff of 335 µmol/L. To illustrate the assessment of agreement between HbA1c and %GA, a difference plot was designed with standard deviations from the results (Figure 2).

**Figure 1.**
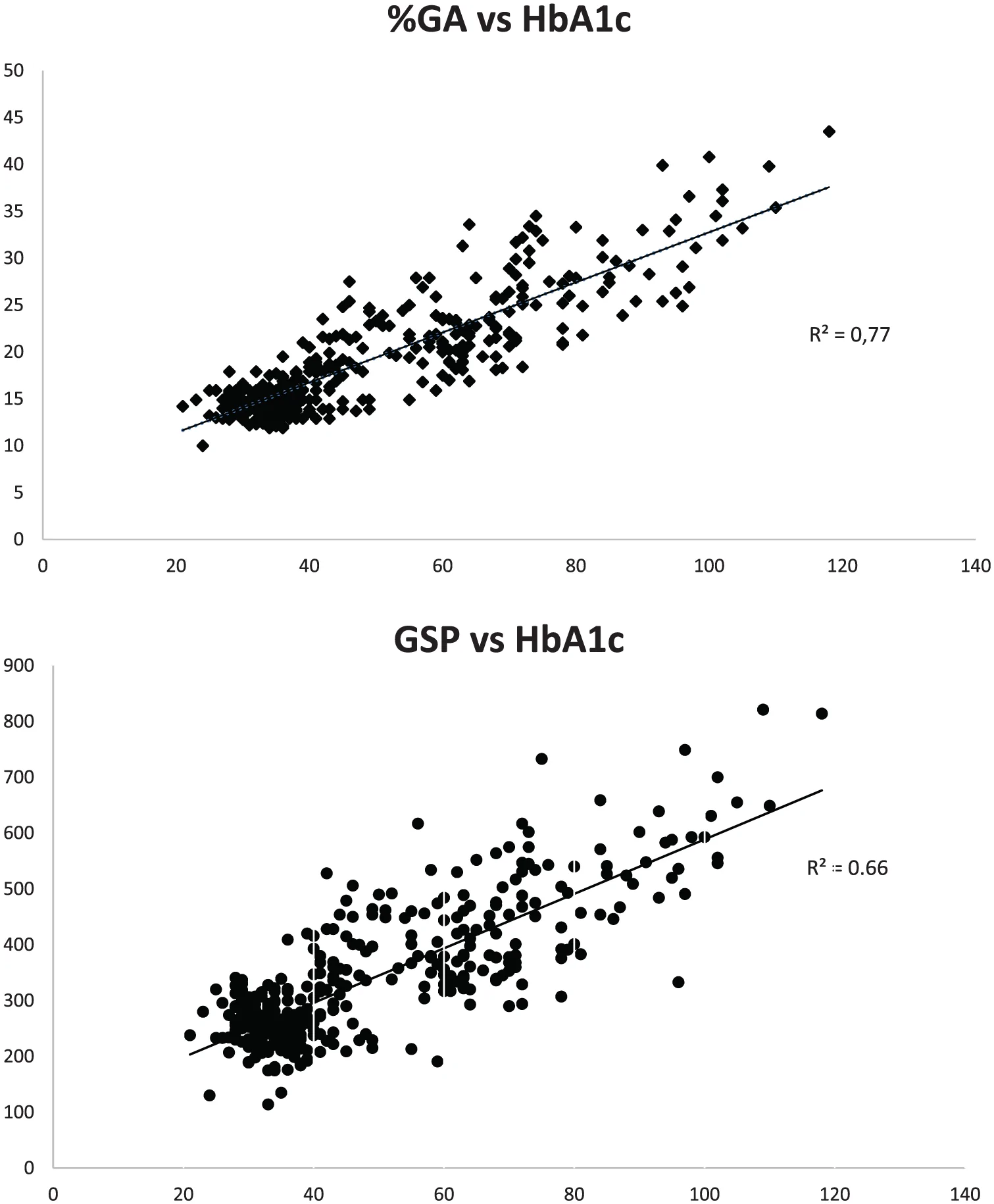
Upper chart: The %GA correlation to HbA1c (mmol/mol) (y = 0.267x + 6.04 R² = 0.7704). Lower chart: Correlation between GSP (µmol/L) and HbA1c (mmol/mol). The GSP correction for total albumin concentration (y = 4.8771x + 100.93 R² = 0.6551).

**Figure 2.**
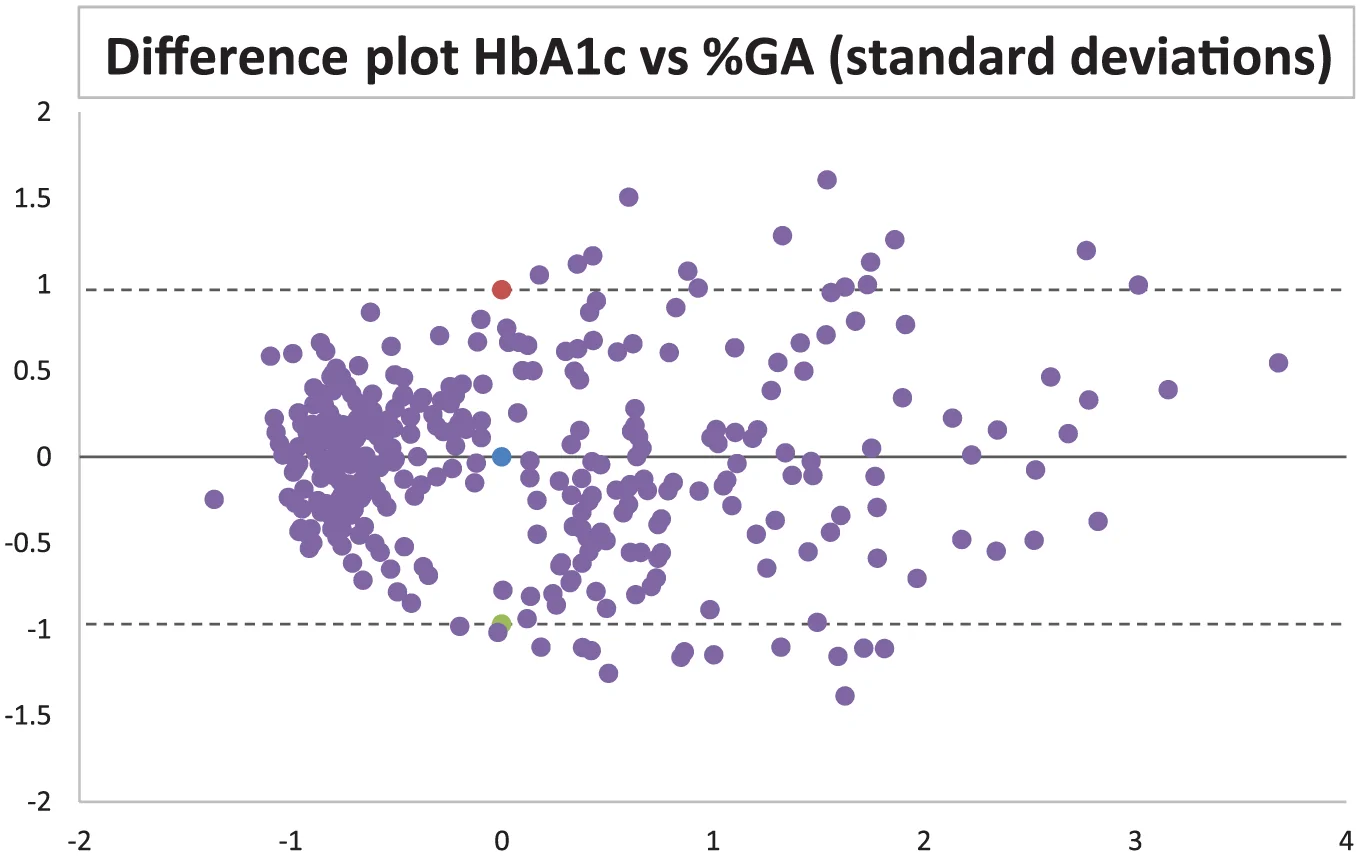
Difference plot/Bland Altman plot using the Z scores of HbA1c and %GA. X axis shows mean value between the two variables and Y axis shows the difference.

### Age and Gender

Overall, the males (n = 157) had mean 20.1 %GA and mean HbA1c 53.8 mmol/mol. Females (n = 114) had mean 19.1 %GA and mean HbA1c 51.0 mmol/mol. When divided into a group of younger (5-70 years) males (n = 103), they had mean values of 19.0 %GA and HbA1c 52.0 mmol/mol while females in the same age group (n = 74) showed mean values of 17.9 %GA and HbA1c 46.8 mmol/mol. Among the oldest (>70 years of age) the males (n = 53) had mean values of 22.3 %GA and HbA1c 57.0 mmol/mol and females (n = 40) had mean values of 21.3 %GA and HbA1c 58.7 mmol/mol. Statistically significant differences were seen between younger and older females regarding %GA (*P* = .014) and HbA1c (*P* = .006). Statistically significant difference was also seen between younger and older males as regard %GA (*P* = .021).

Coefficients of determination were calculated in different cohorts. Younger and older females (5-70 respectively >70 years of age) had *R*^2^ of 0.76 in both cohorts (95% CI were 0.72-0.80 and 0.69-0.83, respectively) while among younger and older males (5-70 respectively >70 years of age), *R*^2^ were 0.83 (95% CI 0.77-0.89) and 0.78 (95% CI 0.71-0.85), respectively.

### High and Low

We also divided the samples into two groups based on their HbA1c result. One group with high HbA1c levels (n = 101) and one group with low HbA1c levels (n = 103). The cut offs were set to >56 mmol/mol and <40 mmol/mol, respectively. The group of high HbA1c levels samples was further divided into subgroups (Figure 3). Statistically significant differences in %GA were observed between all the assessed subgroups.

**Figure 3.**
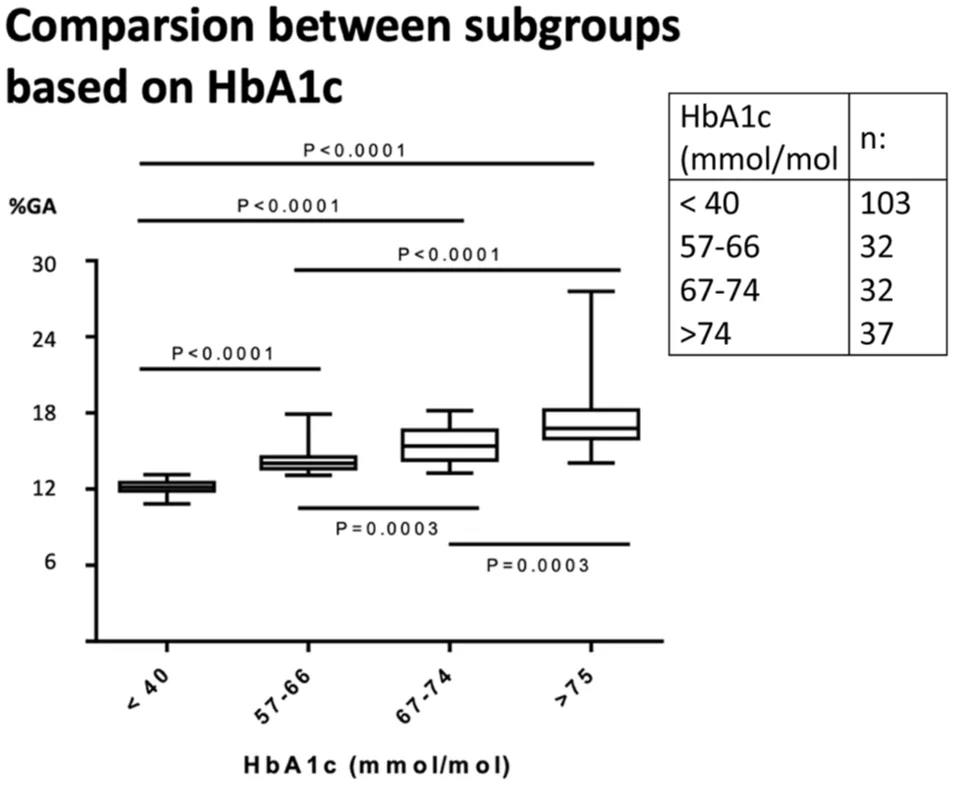
Low HbA1c samples (column 1) and high HbA1c levels samples based on HbA1c-result were divided into subgroups. Statistical significant differences were seen between all the different subgroups for %GA.

## Discussion

Glycated albumin is considered the gold standard for monitoring long-term glucose levels in our routine health care system, and an important factor affecting treatment in patients with diabetes mellitus. It has also been established as a marker for the diagnosis of diabetes mellitus. However, the limitations of using HbA1c are obvious in conditions where the erythrocyte turnover is altered but also in patients in hemodialysis therapy and hyperglycemic conditions with a relatively rapid onset, such as gestational diabetes mellitus and also diabetes mellitus type 1.^8,9,15^ The %GA analysis has, with various methods, been shown to have a good precision and an excellent correlation with HbA1c, even though concerns have been raised concerning decisions limits.^16^

In this study, we found a strong correlation between HbA1c and %GA in different groups based on gender and age. The variability in %GA was somewhat higher in patients with high HbA1c values (HbA1c > 74 mmol/mol). This may be explained by that this group of patients possibly fluctuate more in glucose levels over time. The latter assumption could explain the discrepancy between the two analyses since they monitor analytes with very diverse life span in the human blood circulation.

The age range of the study samples (5 and 95 years) reflects a selection of individuals monitored for long-term glucose status and for diabetes mellitus. Even though a few persons under 18 years of age (n = 6) were included in the study, the absolute majority of included persons were middle aged and older (the total mean age of included individuals was 62.6 years). We did not have knowledge of the included individuals’ medical history, including the eventual presence of hemoglobinopathies among included individuals, which is a limitation of the study.

Using the calculated equation to link GSP and %GA with HbA1c at the cutoff level 48 mmol/mol (Figure 1) the suggested upper cutoff levels were GSP of 335 µmol/L and %GA of 18.9. This can be compared with previous suggested levels for GSP at a very similar level at 340µmol/L and a slightly more diverted level for %GA at 17.5, but in general, it can be regarded as a very good concordance with that study.^12^

The division of HbA1C into high (>56 mmol/mol) and low (<40 mmol/mol) cohorts was based on obtaining two equal large cohorts. These limits are 8 mmol/mol above and below the recommended cut offs for diagnosis of diabetes mellitus, 48 mmol/mol. In our sample, HbA1C levels of >56 mmol/mol and <40 mmol/mol, were separated by approximately two standard deviations (SD) from the cut off limit of diabetes (48 mmol/mol).

Statistically significant differences were observed between young and old females for both the HbA1c method and the %GA method, while among the same age cohorts among males only a significant difference was observed for %GA. Possible explanations for this can be that among older individuals, a higher proportion has an altered erythrocyte turnover due to diagnoses such as long-term kidney disease and need of hemolysis which could give the %GA method some advantages in following the glycemic status in this population.

The glycated albumin analysis proposed weaknesses is dependent on physiological factors, such as nutrition status, inflammation processes and liver function.^17^ Using the %GA, that is, correcting for the individual albumin value we achieved a better correlation with HbA1c compared with the solitary GSP-method, *R*^2^ 0.77 compared with *R*^2^ 0.66.

The analyses of GSP and fructosamine principally reflect the fraction of total serum proteins that have undergone glycation and compared with this principle there is a theoretically benefit using %GA because of the correction to total serum albumin which is the most abundant serum protein and also the serum protein most prone to fluctuate in between individuals depending on different medical conditions.^9,11^ This suggests that assessing %GA instead of GSP and fructosamine eliminates, at least partly, the principal proposed weakness of the glycated albumin, GSP or fructosamine concept. This strengthens the possibility that %GA could be used as a possible alternative or at least a supplement to the HbA1c-method in earlier mentioned patient populations.

Further studies that investigate if %GA, in addition to HbA1C, can provide supplemental information on long-term glucose exposure in subjects with diseases, such as long-term kidney disease, hemoglobinopathies and gestational diabetes are warranted. Further work on reference ranges is also suggested.

## Conclusion

In this study, we found a strong correlation between %GA and HbA1c which indicates that this analysis may be useful in some clinical contexts. In particular, it could become a useful supplement to HbA1c when monitoring the glycemic status in patients with an altered erythrocyte turnover. This includes conditions as some hemoglobinopathies, severe long-term kidney disease and other conditions with an obvious hemolysis. Another group where %GA could provide potential useful information is gestational diabetes and in patients with a relatively rapid onset of diabetes mellitus. Further studies investigating the potential value of %GA in these patient groups is needed.
